# Social Inequalities in Young People's Mental Distress During the COVID-19 Pandemic: Do Psychosocial Resource Factors Matter?

**DOI:** 10.3389/fpubh.2022.820270

**Published:** 2022-03-14

**Authors:** Ingrid Schoon, Golo Henseke

**Affiliations:** ^1^Social Research Institute, Institute of Education, University College London, London, United Kingdom; ^2^Institute of Education, University College London, London, United Kingdom

**Keywords:** social inequalities, mental health, young people, optimism, self-efficacy, social support, COVID-19

## Abstract

The COVID-19 pandemic disproportionately affected young people aged 16–25 years and has brought about a major increase in mental health problems. Although there is persisting evidence regarding SES differences in mental health status, there is still little knowledge of the processes linking SES to young people's mental health, in particular during the current pandemic. Guided by a stress process model this study examines the role of different psychosocial resource factors in mitigating the vulnerability to mental distress among disadvantaged young people and specifies a range of possible mediating pathways. The research draws on a nationally representative longitudinal sample of 16–25 year-olds who participated in the Youth Economic Activity and Health (YEAH) online survey conducted in the UK between February and October 2021. Mental health was measured using the Hopkins Symptom Checklist for anxiety and depression. Socio-economic disadvantage was indicated by parental education and receipt of free school meals. Experience of stress was indicated by financial strain. Psychosocial resource factors included indicators of optimism, self-efficacy, and social support. We controlled for age, gender, living arrangements, and economic activity of the young person (being in education, employment or NEET). The findings suggest sequential mediating processes where SES influences are partially mediated *via* financial strain and the psychosocial resource factors. In addition, the psychosocial resource factors showed independent effects supporting mental health in the face of socio-economic adversity. Moreover, social support played a significant role in boosting self-efficacy and optimism, which in turn reduce mental distress. The findings highlighting the importance of specifying the assumed mediating processes, and to consider multiple resource factors instead of single aspects to gain a more comprehensive understanding of the processes linking SES to young people's mental health.

## Introduction

Young people have been hit particularly hard by the COVID-19 pandemic: their education has suffered (1), their employment prospects are increasingly uncertain (2, 3), and there has been a massive rise of mental health problems (4–7). The pandemic has exacerbated a mental health crisis which already disproportionally affected young people (8–10). The onset of mental illness across the lifespan is highest among children and young people, with 70% of cases emerging before age 24 (11). Mental health conditions developing in early life have a high risk of persisting at later ages and are predictive of a range of negative social and economic outcomes, including educational attainment, employment, and adult health (12, 13). The lifetime costs of poor mental health highlight the potential return on investment of intervention in this age group, as they could help to improve the education and employment status of youth and their subsequent life chances (14).

While all children and young people can experience mental ill-health, there is a persistent association between social inequality and mental illness (12, 15, 16). Children and young people from a relative disadvantaged socio-economic status (SES) background have a higher risk of experiencing mental health problems than their more privileged peers (17–19). The mechanisms linking family SES to mental health are however less well understood (20–22). Multiple mechanisms may contribute to the association between SES and mental health, including material deprivation, lack of access to health services, discriminatory experiences, cumulative stress exposure, and the inability to meet chronic stress with relevant resources to help to reduce its psychological and biological impact (22–24). The focus of this study is the latter pathway, examining the availability of psychosocial resources and their effectiveness in reducing the negative impact of SES differences on young people's mental health.

Individuals facing socio-economic adversity bring with them a range of psychosocial resources (e.g., self-efficacy, optimism, and social support) that can potentially reduce the impact of adverse experiences. Exploring the role of a range psychosocial resource factors (PRF) in mitigating the vulnerability to mental distress among disadvantaged young people, this study aims to gain a better understanding of the mechanisms linking SES to young people's mental health and the identification of possible protective factors. In particular, we will assess (a) the strength of the association between SES, PRFs, and mental distress; and (b) the role of different PRFs in protecting the mental health of young people experiencing socio-economic adversity. We focus on young people aged 16–25 years in the UK, since levels of wellbeing among children and young people in England and the UK remain relatively low compared with other high-income countries (25), and there are relative high levels of income inequality (12).

## The Stress Process Model

According to the highly influential stress process model (23, 26, 27) differences in people's health and wellbeing correspond to differences in their SES locations. SES does not necessarily directly impact individual health, but may, instead, exert its effect indirectly through the differential exposure to different demands and social stressors. Social stressors refer to a broad array of adverse conditions and experiences, such as precarious employment and living conditions or economic strains, that can challenge the adaptive capacities of individuals. Notable, social stressors can reflect either the experience of prolonged hardship, chronic strain and daily hassles or the experience of disruptive events, such as losing one's parent, or sudden changes to household income, which in turn can proliferate pre-existing strains (23, 28).

Social stress processes can start in childhood, as persistent and recurrent exposure to hardship can impact children's bodily systems and brain development (26, 29), which, in turn, can influence the development of PRFs (such as self-concepts or perceptions of mastery) needed to cope with negative experiences. Moreover, the stress process model and the related reserve capacity model (30) argue that SES can shape appraisals of one's circumstances in a way that further increases stress burden. Both models, build on the assumption that individuals with lower SES are exposed to more stressful experiences and thus have fewer opportunities to develop crucial psychosocial resources that enable them to manage effectively positive and negative experiences related to health status and contextual stressors. A decreased ability to deal with stress in response to recurrent stressors then leads to increased susceptibility for psychological and physical distress, including anxiety and depression.

The intensity of the stress that people experience cannot be adequately predicted from the intensity of the stressors alone. Instead, people typically confront stress-provoking conditions with a variety of PRFs that can moderate perceptions of the adverse conditions or mediate their impact.^1^ These PRFs typically include both intra-personal resources such as self-efficacy and optimism as well as inter-personal resources such as social support (23, 27, 30). Given variations in these PRFs there can be substantial variation in how individuals in similar circumstances respond to the same event or circumstances.

The major conceptual elements of the stress process model are the sources of stress (the social stressors), the manifestations of stress (e.g., anxiety and depression), and the PRFs that can be invoked by people on behalf of their own defense and serve as moderators or mediators of stress. An advantage of stress process models is their ability to clarify the complex relationship between SES and health outcomes and to identify relevant PRF and behaviors that are malleable through interventions. The role of PRF as potential mediators of SES effects on mental health has been confirmed in a number of studies and approaches (22, 24, 27, 31), in particular regarding the role of self-efficacy, optimism and social support. There is however still a lack of understanding regarding the pathways involved, the relative strength of the different PRFs, and their synergistic effect in mitigating stressful SES effects (32). Moreover, while most previous studies have focused on the role of PRFs as mediators or moderators of the SES influences on mental health, there is less understanding regarding the role of SES in shaping PRFs (22, 26). For example, a number of studies report relative small or weak associations between SES and key PRFs (24, 32, 33). Does SES directly influence the manifestations of PRFs; are SES effects mediated *via* social stressors; what is the size of SES influences on PRFs? Moreover, are certain PRFs more effective than others in protecting mental health in the face of SES inequalities, i.e., what is their relative influence when considering multiple PRFs simultaneously? The aims of this study are to specify the pathways linking SES to the experience of social stress (in particular the experience of pandemic related financial strain) as well as different PRFs (focusing on the role of social support, optimism and self-efficacy) and mental distress. Before specifying potential pathways linking SES to mental distress, we provide a definition of SES and its assessment among young people.

### Measuring SES in Young People

SES has been operationalized in a variety of ways (15, 17, 18). Depending on discipline, the focus had been on parental education, income poverty or social class, or a combination of these factors. Despite being positively correlated, past research has shown that parental education, social class, and poverty relate to different forms of parental resources, such as informational, socio-cultural and economic resources—each of which has independent and distinct effects on individual lives (34, 35). For example, better educated parents might help their children to develop skills and strategies to deal with problems effectively and thus raise their perceptions of control, and sense of self-efficacy (36); and a family's financial situation can impact on young people's optimism and outlook to the future (37) because their lives are more predictable and stable. Composite SES measures, such as the Hollingshead Four Factor Index (38), summarize or combine information from multiple SES indices, yet reduce the amount of information available for analysis (39). In any case, unless multiple indicators of SES are considered, there is potential bias in overestimating the effect of a single indicator and underestimating the total effect of family SES (26, 34).

Another issue to be considered here is that assessing family SES among young people, in particular adolescents, can be difficult as they might not know, or not be willing to reveal such information (40). In particular questions regarding parental occupation have shown to be difficult, resulting in low response rates and potential bias (41). An alternative approach utilizing information on household circumstances indicating a measure of family affluence, such as car ownership and housing tenure, has therefore been developed as a useful alternative (40). However, there is also evidence to suggest that young people develop a more stable and accurate sense of their social position as they move through adolescence (42). As the focus of this study is on 16–25 year olds we assume that the knowledge of their social position becomes more accurate, and ask them to report on their parental education and their own eligibility for FSM (FSM), widely used indicators of family SES (43).

### Processes Linking SES Influences to Mental Health: The Role of PRFs

SES does not necessarily directly impact on mental health. One mechanism of how it can effect people is through the experience of economic strain (23). Individuals in relative disadvantaged SES locations might find it difficult to make ends meet, to pay for regular bills, or cannot afford necessities, in particular in the current uncertain economic climate (2). The perception of financial strain, in turn, can impact the development or maintenance of psychosocial resources, such as social support, optimism or self-efficacy, needed to cope with the adverse situation. All of these factors might have a direct effect on mental distress. In addition to direct SES effects on mental health, there can thus also be mediated effects, either through the experience of financial strain and/or through the available psychosocial resources.

In modeling terms, financial strain and PRF are understood as intervening variables. They act as the conduit for some of the influence of SES on mental health because they are influenced by SES, on the one hand, and because they influence mental health, on the other. Intervening variables are essential components of mediating models, operationalizing the mechanisms that connect SES to mental health outcomes. For example, there is evidence to suggest that optimism is associated with both family SES and depression (32, 44). Optimism refers to a positive expectancy about future events and has been consistently associated with the experience of more positive and less negative emotions when faced with a difficult situation across a wide variety of contexts, including health problems (45, 46). In addition, optimism has been positively associated with approach coping strategies and negatively associated with avoidance coping strategies (47). Other crucial PRFs, including mastery (or self-efficacy) as well as social support have shown to mediate the association between SES and depression (26, 27). Self-efficacy, i.e., people's belief that their actions can actually have a positive impact on the world, is central to effective functioning and is associated with a range of developmental outcomes in later life including mental health (48). Also the benefits of social support for maintaining mental health during the COVID-19 pandemic have been widely recognized (49, 50). Moreover, there is evidence to suggest that social support shapes the development of other person-level resources, such as optimism (32, 51). There is thus a range of possible mediating processes linking SES to mental health. Moreover, there could be potential independent effects. Given that a number of studies report relative weak associations between SES and PRFs (26, 33), it might be the case that psychosocial resources are associated with positive mental health independent of parental SES.

**Figure 1:**
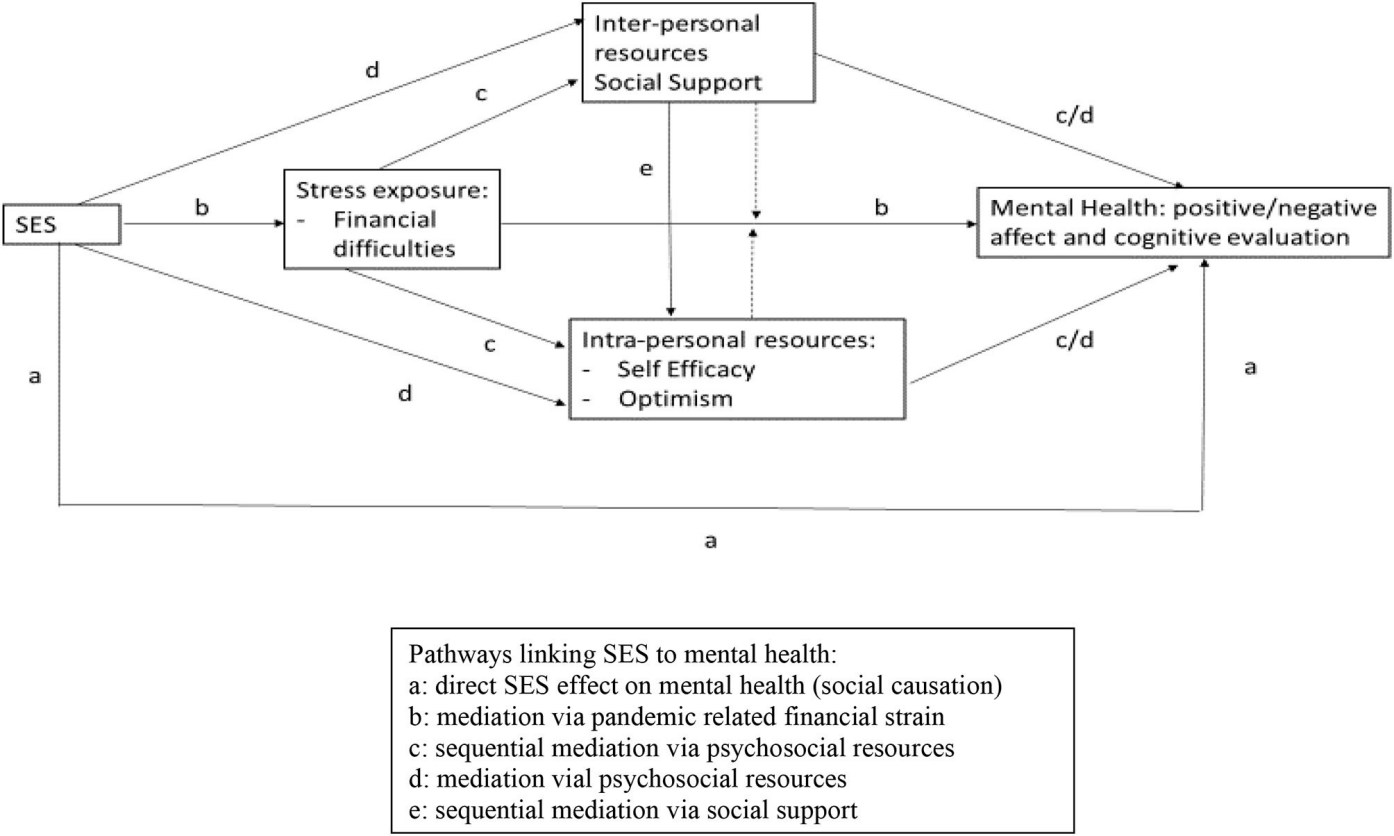
Sequential mediating processes linking SES, financial stress, psychosocial resource factors, and mental health.

Figure 1 gives an overview of possible pathways, including:

a *Direct SES effect* on mental health (path a in Figure 1). This pathway assumes that SES influences mental health directly, without mediation.
b *Mediation via financial stress*. The stress process model (23, 27) argues that SES influences on mental health are not necessarily direct (path a in Figure 1) but are mediated by experiences of stress, in this case pandemic related financial strain. To examine if SES influences on mental health are mediated *via* financial stress we assess the associations between family SES and financial strain (path b in Figure 1) as well as subsequent mediated influences on PRFs and mental health (paths c), suggesting sequential mediating processes.
c *Mediation via PRFs*. The stress process model (23) as well as the resource capacity model (30) suggest that SES influences on mental health are mediated *via* psychosocial resources. There can be direct SES influences on the level of psychosocial resources, which, in turn, fully mediate SES influences on mental health (see paths d in Figure 1). Such a process would indicate cumulative processes of resource amplification (52), where for example low SES is associated with low levels of PRFs and high levels of mental distress. As already mentioned, there might also be the case of sequential mediation *via* financial stress, which, in turn, impacts on psychosocial resources, which in turn impact on mental health (paths c in Figure 1). Moreover, the dashed lines indicate potential moderator effects, i.e., a reduction of the association between financial strain and mental health by the resource factors.
d *Sequential mediation via social support*. The stress process model (23) also suggests that experiences of social support can potentially buffer against the effects of economic strain by sustaining self-concepts and mastery in the face of persistent strain and thereby inhibiting depression (path e in Figure 1). A similar model has been tested by Zou et al. (32), who showed that higher SES predicted greater social support and increased optimism, which in turn contributed to reduced depression.

## The Current Study

This study assesses the viability of an elaborated stress process model, testing different assumed pathways linking SES to mental distress. We expect that SES is associated with the experience of financial strain and a range of PRFs, which in turn are associated with mental distress. As indicated in Figure 1 we will test for possible (a) direct effects of SES on mental health without mediation; (b) mediation *via* financial stress; (c) mediation (including sequential mediation) *via* PRFs; and (d) independent effects *via* PRFs. We expect that (H1) SES is positively associated with (a) financial strain; and negatively with (b) PRFs; and (c) mental distress. (H2) financial strain mediates SES influences on mental distress either (a) directly or (b) *via* sequential mediation through optimism, self-regulation and social support. (H3) optimism, self-regulation and social support mediate SES influences on mental health (no sequential mediation *via* financial strain). (H4) social support may facilitate the maintenance of self-regulation and optimism despite experience of financial strain, suggesting another process of sequential mediation. (H5) there are independent effects of the PRF, over and above the influence of SES and financial strain on mental distress.

## Methods

### Data and Sample

The study is based on the first four waves of the Youth Economic Activity and Health (YEAH) Monitor, a quarterly quota panel study of 16–25-year-old UK residents with 1,000 observations per wave recruited from web access panels managed by Ipsos Mori and partners. For the initial sample, quotas were set according to age within gender, working status and region. Follow-up samples were recruited among previous participants when possible and refreshed according to the quotas to make up for attrition when necessary. In conjunction with supplied survey weights, the sample was designed to be nationally representative. The longitudinal response rates were 46% for wave 2 and 51% for waves 3 and 4. On average, respondents contributed to 1.47 survey waves. The following analyses are based on 2,402 respondents, using complete cases. We report pooled findings for waves one (January/February 2021), two (April/May 2021), three (July 2021), and four (October 2021). The study has full ethical approval from the UCL IOE Research Ethics Committee and is registered with the UCL Data Protection Office (Z6364106/2020/10/90).

### Measures

#### SES Exposure

Socioeconomic status is measured by two categorical indicators comprising parental education and eligibility for FSM. Study participants reported on their parent's highest level of education. We distinguish between parents with little or no formal education (1), (upper-) secondary level attainment as measured by the General Certificate of Secondary Education at grades A-C or equivalent or A-level or equivalent (2), and those who achieved tertiary qualifications (3). Study participants were also asked whether “Whilst at school in the UK, have you EVER received free school meals?” Eligibility for FSM is a widely used indicator of SES (43).

#### Social Stress (Financial Strain)

To assess subjective perceptions of pandemic-related financial strain, study participants were asked: “All things considered; how well would you say you yourself are managing financially these days?” Responses were coded on a 5-point scale, ranging from living comfortably (1), doing alright (2), just about getting by (3), finding it quite difficult (4), to finding it very difficult (5). This indicator thus relates to the financial strain experienced by the young person, not the parents.

#### Mediating Factors

We used three indicators of PRFs, including optimism, self-efficacy, and social support. *Optimism* was assessed using the Perceived Life Chances Scale (53), a 10-item scale asking respondents “Thinking about how you see your future, what are the chances that...,” with response items including, for example, “You will have a job that pays well,” “You will have a happy family life,” “You will be respected in your community,” and “Life will turn out better for you than it has for your parents,” rated on a scale from 1 (very unlikely) to 5 (very likely) with an explicit option for don't know. We dichotomized the responses to distinguish between those who deemed positive life outcomes likely and those who did not. Previous research has established the scale as a valid predictor of psychosocial adjustment of young adults with good internal consistency (54). *Self-efficacy* is assessed using the 6-item short form of the General Self-Efficacy Scale (GSE-6): a valid and reliable assessment of general self-efficacy (55–57). Respondents are asked to rate on a 5-point scale the degree to which they agree with statements such as: “I can solve most problems if I put in the necessary effort,” “I can remain calm when facing difficulties because I can rely on my coping abilities,” “It is easy for me to stick to my aims and accomplish my goals,” “No matter what comes my way, I'm usually able to handle it.” *Social support* is assessed with a single-item measure, asking “How many people, if any, do you have with whom you can discuss intimate and personal matters?” Responses ranged from 0 (none) to 10+. The item was adopted from the European Social Survey (58).

#### Outcome: Mental Distress

Mental distress was assessed using a short-form Hopkins Symptom Checklist (HSCL-5), a five-item scale designed to yield a brief evaluation of psychological well-being in terms of worry, anxiety and dysphoria in general population surveys (59). HSCL-5 has shown good reliability as a measure of psychological distress with satisfactory construct validity (60, 61). The instrument asks respondents to report how much they were bothered by feelings of fearfulness, nervousness, hopelessness, sadness and worries in the week before the interview, with responses ranging from 1 “Not at all” to 4 “Extremely”.

#### Control Variables

To account for possible alternative explanations, in particular regarding the wider structural context, the estimation models control for a range of time-variant and time-invariant socio-demographic control variables. These include age (continuous measure in years), gender (male vs. female), ethnicity (white vs. other), current economic activity (differentiating between those in education, employment or NEET), living arrangements (living with parents or not), and survey months. Previous research has shown persistent gender differences in mental health, and about twice as many women experience major depression than men among adult clinical and community samples (62). Moreover, there are ethnic differences, and people of color are in general at a greater risk of stress exposure and socio-economic hardship in particular during the COVID-19 crisis (63, 64). However, among adolescents in the UK research evidence suggests better mental health and wellbeing among ethnic minority adolescents than whites (65–67). These findings point toward the so-called “race mental health paradox”, i.e., that despite higher stress exposure and greater material hardship ethnic minorities tend to report similar of relatively lower levels of mental health problems than whites (68, 69), which is also observed among adults (70). In addition, we account for variations the economic activity of young people themselves. The period between age 16–25 marks a transition period characterized by the assumption of new social roles, with the transition from education to employment being a key transition marker with implication for mental health status (8). While the majority of 16–18 year olds in the UK are continuing in education, by age 20 about half of all young people have entered the labor market (71). Generally, education and employment can offer young people an important opportunity to fulfill psychological needs and develop financial independence, agency, and a positive social identity (72, 73), although employment in this age group has been defined by increasing precarity. Young adults not in employment, education or training (NEET) report the worst mental health outcomes (74). Living with parents could help to alleviate stress, for example, through resource sharing or additional social support (75). However, living with parents might also exacerbate stressors if young people had little control over their circumstances or negative family experiences due to lockdown (76). Controls for survey months condition out seasonality, changes in the pandemic threat or the lifting of lockdown restrictions on individual behavior as vaccine became available that might otherwise shift mental health and reported psychosocial resources.

#### Analytical Strategy

To assess the relationship of SES with mental distress and the potential intervening role of financial strain and PRFs, we use linear Structural Equation Modeling (SEM) as implemented in STATA 17 in the pooled sample. SEM enables us to simultaneously estimate the structural relationships of socio-economic background with stress, PRFs, and mental health. The models yield estimates of (i) the direct, conditional, relationship of SES with the mediator variables (financial strain, PRFs) and mental distress as well as (ii) the total, unmediated, effect of SES on mental distress. To test the earlier discussed hypotheses, we assess whether SES is significantly associated with the proposed mediator variables and whether it correlates with mental distress. The extend of mediation can be gleaned from a comparison of the direct, mediated SES influences on mental distress compared to the total effect. If SES influences are fully mediated, they approach zero conditional on the mediators (77, 78).

SEM measurement models were used to derive continuous latent variables of mental health (HSCL-5) and resource factors (optimism, general self-efficacy). The sample includes repeated observations within individuals and is thus unlikely to meet the joint normality assumption of standard SEM. To account for potential autocorrelation within individuals over time, we compute cluster-robust standard errors using a quasi-maximum likelihood approach. Estimates use the supplied survey weights to correct potential sampling bias. Error terms are permitted to correlate. Overall model fit is assessed using the coefficient of determination (*R*^2^) and standardized root mean squared residual (SRMSR). This is an observational study and makes no causal assertions. Terms such as “effect” are used in a purely statistical sense.

## Results

Table 1 shows the correlations between the variables included in this analysis based on the SEM measurement model estimates of the latent constructs. The correlations range between 0.01 and 0.48 and show the expected signs. The largest correlations were between self-efficacy, optimism and mental health. In all, there is no evidence to suggest multi-collinearity. The summary statistics of all variables included in the model are given in Appendix Table 1.

**Table 1:** Correlation table for the model variables SES, financial strain, psychosocial resource factors and mental distress (*N* = 2,402).

	**HSCL-5**	**Future optimism**	**General self-efficacy**	**Social support**	**Financial strain**	**Low parental education**	**High parental education**	**Free school meal**
HSCL-5	1							
Future optimism	−0.260^***^	1						
General self-efficacy	−0.363^***^	0.478^***^	1					
Social support	−0.085^**^	0.171^***^	0.154^***^	1				
Financial strain	0.255^***^	−0.232^***^	−0.225^***^	−0.032	1			
Low parental education	0.010	−0.086^**^	−0.067^*^	−0.087^**^	0.049^*^	1		
High parental education	0.052	0.075^**^	0.104^***^	0.078^**^	−0.085^***^	−0.334^***^	1	
Free school meal	0.155^***^	0.003	−0.017	−0.010	0.136^***^	0.080^**^	−0.081^**^	1

*Estimated linear correlation coefficients. Standard errors adjusted for repeated observations within individuals*.

* *p < 0.05*.

** *p < 0.01*.

*** *p < 0.001*.

**Figure 2:**
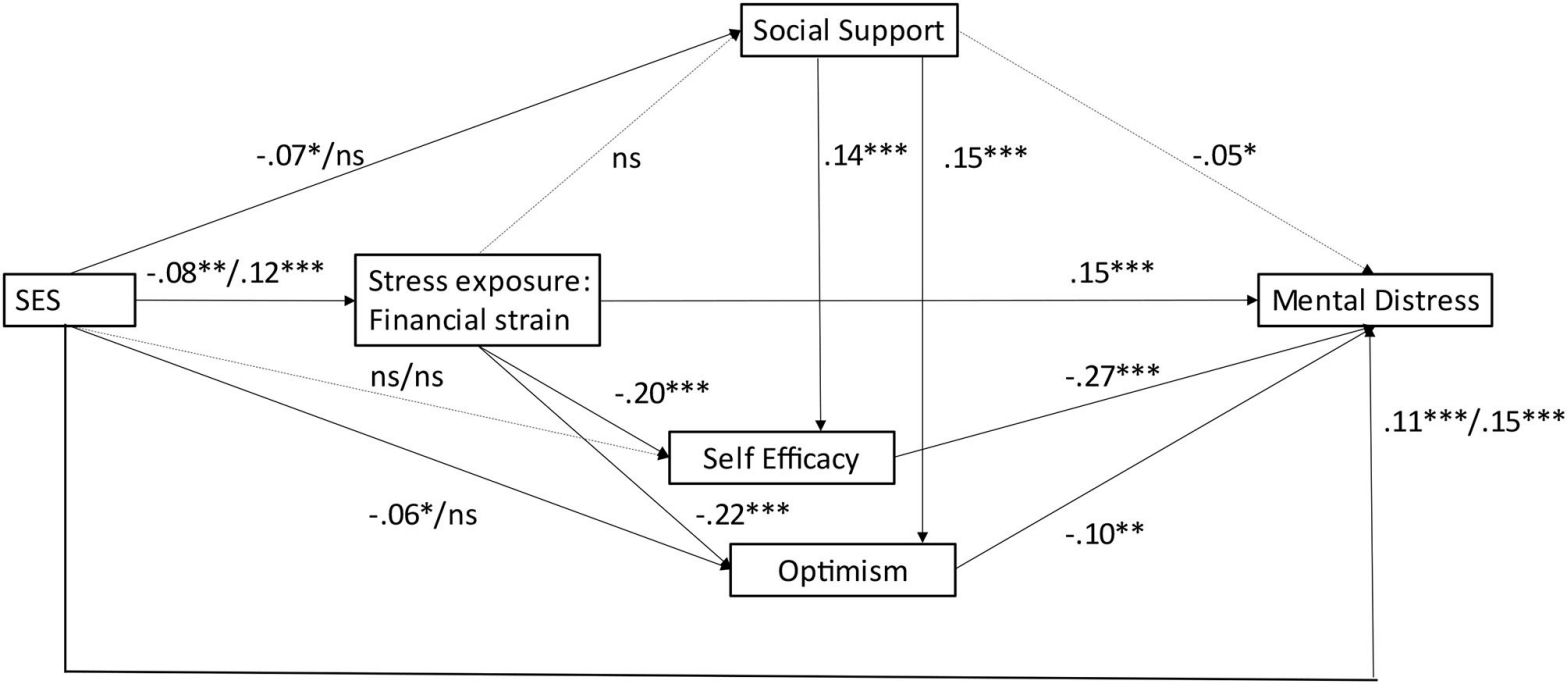
Pathways linking SES, financial strain, psychosocial resource factors and mental distress (SEM estimates). SES effects are shown as: parental education/FSM; dotted lines are non-significant (ns). *p < 0.05, **p < 0.01, ***p < 0.001.

Figure 2 shows the significant pathways linking SES to financial strain, the PRFs, and mental distress (including all controls). Table 2 gives information on the direct and mediated pathways linking SES, financial strain, PRFs and mental distress in a combined model (columns 1–5) and the total effect (column 6). The models include all control variables. The SRMR index is within the acceptable range between 0 and 0.08. The measurement models for the latent variables (see Appendix Table 2) show satisfactory properties across outcomes—most correlations coefficients between the predicted latent variables and the observed indicators were in the range from 0.60 to 0.71. None of the bivariate correlations were below 0.40.

**Table 2:** Pathways linking SES, financial strain, psychosocial resource factors and mental distress (*N* = 2,402, Overall R-Sq. +0.21, SRMR = 0.031).

	**(1) Financial strain**	**(2) Social support**	**(3) General self-efficacy**	**(4) Future optimism**	**(5) HSCL-5**	**(6) HSCL-5**
					**Direct**	**Total**
**Parental education (ref: medium)**						
Low	0.009 (0.024)	–0.065^*^(0.027)	−0.023 (0.031)	–0.057^*^(0.027)	0.009 (0.024)	0.029
High	–0.077^**^(0.026)	0.044 (0.026)	0.056 (0.030)	0.021 (0.028)	0.114^***^(0.026)	0.075^**^
Free school meal (Ref: none)	0.118^***^(0.024)	0.001 (0.024)	0.003 (0.032)	0.028 (0.027)	0.152^***^(0.028)	0.175*^**^
Financial strain		−0.029 (0.025)	–0.204^***^(0.029)	–0.220^***^(0.024)	0.151^***^(0.025)	0.231*^**^
General self-efficacy					–0.269^***^(0.035)	−0.269*^**^
Future optimism					–0.102^**^(0.031)	−0.102^**^
Social support			0.139^***^(0.027)	0.150^***^(0.025)	–0.047^*^(0.024)	−0.100^***^
Control variables	X	X	X	X	X	
R-sq	0.060	0.024	0.107	0.103	0.247	

*Standardized coefficients from a joint structural equation model with measurement components for latent variables (HSCL5, Future Optimism, General Self-Efficacy) using quasi maximum likelihood estimations. All error terms permitted to correlate. Control variables comprise economic status (in education/in work/not in education or work), gender (male/female), age (16–18/>18), ethnicity (white/bame/refused), living with parents (yes/no), and survey wave (wave 2/3/4). Clustered standard errors in parentheses*.

* *p < 0.05*,

** *p < 0.01*,

*** *p < 0.001*.

The findings suggest SES inequalities in the experience of financial strain, the PRFs and mental distress (columns 1–5), partially supporting H1. We find support for H1a, as financial strain is negatively associated high parental education (β = −0.077, *p* = 0.003), and positively with receipt of FSM (β = 0.118, *p* ≈ 0.000). H1b is partially supported, as low parental education is negatively associated with social support and optimism (β = −0.065, *p* = 0.015; β = −0.057, *p* = 0.032) but not with self-efficacy. There are no significant associations between the PRFs and high parental education and FSM. H1c is also partially supported. We find a positive association between FSM and mental distress (β = 0.152, *p* ≈ 0.000), while high parental education shows an unexpectedly positive association with negative affect (β = 0.114, *p* ≈ 0.000).

There is also evidence in support of H2 suggesting that financial strain mediates SES influences on mental distress both directly (H2a) and through sequential mediation *via* self-efficacy and optimism (β = −0.204, *p* ≈ 0.000; β = −0.220, *p* ≈ 0.000) (H2b), but not *via* social support. The hypothesis of mediation of SES influences *via* PRF (H3) is partially supported. There are weak negative associations between low parental education, social support and optimism. However, the SES indicators are not significantly associated with self-efficacy. In addition, we find support for H4, suggesting that social support plays a significant role in boosting self-regulation and optimism in the face of socio-economic adversity.

Generally, the extend of mediation is not very large, given that the direct effects (column 5) do not differ greatly from the total effects (column 6). For example, the total and direct effects associated with parental education and FSM are fairly similar. We see the largest differences for mediation of financial strain *via* the PRFs, although the direct effect is still different from zero. Moreover, we find significant independent effects of the PRFs (H5), in addition and above the influence of SES and financial strain on mental distress. The strongest (negative) associations are observed between self-efficacy and mental distress (β = −0.269, *p* ≈ 0.000), followed by optimism (β = −0.102, *p* ≈ 0.000) and tendentially also social support (β = −0.047, *p* = 0.047).

Additional analysis (Appendix Table 3) considers the impact of the control variables on financial strain, PRFs and mental distress. The experience of financial strain is significantly higher among those in education or NEET (compared to those in employment) and among ethnic minority youth, while those living with their parents or legal guardian report lower financial strain. Perceived social support is low among those who are NEET, who live with their parents, and males. General self-efficacy is lower among those who are NEET or live with their parents and is higher among males than females. Future optimism is lower for those in education or who are NEET and those who live with their parents, and higher among those aged 16–18 (compared to those who are 19–25). Mental distress is higher among those living with their parents, among males and ethnic minority youth and lower among 16–18 year olds (compared to those aged 19–25).

Generally, standardized SES coefficients (and those of the controls) on resource factors and mental health indicators are only small, ranging from −0.103 to 0.188. The findings suggest partial mediation of SES effects *via* financial strain. Moreover, there is evidence for sequential mediation *via* psychosocial resources, in that financial strain is associated with psychosocial resources, in particular self-efficacy and optimism, which in turn are associated with lower levels of mental distress. In addition, we find sequential mediation *via* social support, which is negatively associated with low parental education and positively with self-efficacy and optimism. Besides, there are independent protective effects of the PRFs on mental health in addition and above SES influences. Overall, the model explains 25% of the variation in mental distress.

## Discussion

The aims of this study were to assess an elaborated stress process model, specifying a range of direct and mediating processes linking SES to mental distress among 16–25 year olds during the current COVID-19 pandemic. We find social inequalities regarding the distribution of mental health (H1c), but also demonstrate the significant role of PRFs in protecting mental health in the face of socio-economic adversity. The key contribution of this paper is to clarify the pathways linking family SES (indicated by parental education and eligibility for FSM) to social stressors (financial strain), multiple PRFs, and their joint impact on mental distress. Moreover, we assess the relative role of multiple PRFs as mediators of SES influences and predictors of mental health. We find evidence in support of distinct sequential mediation processes, both *via* financial strain and PRFs, as well as independent PRF effects.

SES disadvantage is associated with raised levels of social stress (experiences of financial strain) (H1a), but also is directly associated with mental distress, suggesting that not all SES influences are mediated *via* social stressors or PRFs. Financial strain mediates some SES influences on mental distress (H2a). Sequential mediation is only evident for optimism and self-efficacy (H2b), but not for social support. The assumption that SES influences are mediated *via* PRFs (H3) is only partially supported, as SES indicators only show weak associations with social support and optimism and are not associated with self-efficacy. Our findings are in line with previous studies suggesting that SES is not strongly associated with PRFs (24, 32, 33). Over and above the SES influences we find independent beneficial effects of the PRFs (H5), suggesting that they can to some extent mitigate SES influences on mental health. In particular, self-efficacy as well as optimism play a significant role in reducing mental distress in the face of adversity. Moreover, social support can boost the development of self-efficacy and optimism in the face of hardship (H4), highlighting the vital role of inter-personal resources in promoting positive development (79, 80) even in adverse conditions (32, 51), including the current pandemic.

SES influences are only partially mediated *via* financial strain and PRF, and we do find some support for the assumption of direct SES effects mental health (H1a). In particular, the receipt of FSM is associated with mental distress (and the experience of financial strain). In addition, there is an unexpected positive association between high parental education and mental distress, and the direct effect exceeds the total effect. In other words, higher SES is not invariably associated with lower levels of distress (26). The finding could indicate that there is potentially a greater awareness of or readiness to report mental health problems among high SES offspring and more research is needed to unpack these associations and potential bias in reporting. In any case, taking into account multiple SES indicators creates a better understanding regarding their relative role in shaping mental health of young people during the pandemic.

In addition, considering multiple PRFs instead of only one provides a better understanding of their different and interlinked contributions in promoting mental health despite the experience of socio-economic adversity. Compared to the indicators of self-efficacy and optimism, social support appears to be least affected by SES indicators and experiences of financial strain (as indicated by the lower *R*^2^ in Table 2). Nonetheless, all three PRFs considered here appear to reduce mental distress in the face of socio-economic adversity. Notably, the protective role of social support, comes mainly through its boost for self-efficacy and optimism [see also (23)]. The different PRFs considered here thus seem to have different functions in promoting mental health, with social support being a crucial mediating resource that can be invoked by people to strengthen their defense against stress.

More generally, the findings highlight the relevance of an elaborated stress process model for gaining a better understanding of the multiple and interlinked processes underlying the development of mental health. In addition to guiding the conceptualization of assumptions about possible pathways linking SES to mental health, the stress process model provides a more comprehensive understanding of possible sources and manifestations of stress. According to the principle of stress proliferation (23, 28) sudden events can create new strains or intensify pre-existing stressors. The COVID-19 pandemic and associated social and economic uncertainties might have increased financial strains, further depleted the PRFs and increased mental distress. Future research should examine the pandemic related stressors in more detail.

Regarding the influences of the control variables, the findings suggest that transition events, such as the current economic activity of the young person and their living conditions matter in shaping their resource factors and mental health. In particular those who are NEET report higher levels of financial stress, lower levels of social support, self-efficacy and optimism for the future, suggesting that uncertainties regarding education and the transition into work might have raised the stress levels and reduced psychosocial resources. Also, those living with their parents or legal guardian during the pandemic report higher levels of financial strain, lower levels of PRFs and higher levels of mental distress, suggesting that they might encounter additional strains due to lack of control or negative family experiences due to lockdown (76). The findings also show that ethnic minority youth report higher levels of financial strain but lower levels of mental distress, providing some support for the “race mental health paradox” (65–67). However, this finding could also reflect cultural differences in reporting and the stigma related to mental health.

## Limitations

In interpreting the findings, a number of limitations have to be considered. The online study is largely based on self-reports not observed data. The sample is based only on those young people with access to the internet, limiting the generalization of findings to this group. Family SES was assessed based on reports of young people, which can introduce potential reporting bias. The relatively small sample limits the scope for subgroup analysis to test if the estimated relationships hold across different demographics. The study suggests that men report lower levels of financial strain, less social support, higher levels of self-efficacy, and lower levels of mental distress than women. The findings are in line with existing studies that suggest that young women's mental health was worse affected by the pandemic than those of men (6, 72). It would be worthwhile to examine gender differences in the pathways linking SES to mental health during the COVID-19 pandemic in more detail. Moreover, the short time dimension of the panel limited the analyses to the concurrent association of stressors with mental health outcomes. It is conceivable that the effect of cumulative and persisting stressors could potentially increase social inequalities regarding PRF and mental health outcomes and reduce the beneficial role of psychosocial resources. We did not make full use of the longitudinal nature of the data and future studies should examine intra-individual variations over time in more detail. Finally, the non-random nature of the sample and focus on the UK hampers generalizability to other contexts. Despite these limitations, this study enables a more comprehensive understanding of the processes linking SES, financial strain, PRF and mental health of young people during the COVID-19 pandemic.

## Conclusion

The current study specifies the pathways by which SES impacts on mental health of young people and suggests that PRFs can play a significant role in reducing mental distress in the face of socio-economic adversity. We find sequential mediating processes where SES influences on mental health are partially mediated *via* experiences of financial strain, which depletes psychosocial resources, which in turn, shape mental health outcomes. There are however also direct SES effects on mental health, and independent beneficial effects of the PRFs, suggesting only partial mediation. Psychosocial resources that individuals bring to a stressful situation can help them to cope with this stressful encounter—if the stresses are not overpowering. Moreover, PRFs build on each other. For example, this study has shown that social support can boost the development of optimism and self-regulation even in the face of socio-economic adversity. To promote the mental health of young people it is thus important to reduce SES risk factors and associated strains, and to reinforce the development of PRFs. Future research should examine constellations of multiple SES indicators, multiple PRFs, and different dimensions of mental health in more detail to gain a better understanding of how these combine and interact, in particular in times of high uncertainty that characterizes the current COVID-19 pandemic.

## Data Availability Statement

The raw data supporting the conclusions of this article will be made available by the authors, without undue reservation.

## Ethics Statement

The studies involving human participants were reviewed and approved by UCL Research Ethics. Written informed consent from the participants' legal guardian/next of kin was not required to participate in this study in accordance with the national legislation and the institutional requirements. The study has full ethical approval from the UCL IOE Research Ethics Committee and is registered with the UCL Data Protection Office (Z6364106/2020/10/90).

## Author Contributions

IS and GH contributed to the conception and design of the study. GH organized the database, performed the statistical analysis, and contributed to the different sections, in particular section Methods and Results. IS wrote the first draft of the manuscript. All authors contributed to manuscript revision, read, and approved the submitted version.

## Funding

The authors want to acknowledge funding from the UK Economic and Social Research Council, Grant Number ES/V01577X/1 and ES/T001526/1.

## Conflict of Interest

The authors declare that the research was conducted in the absence of any commercial or financial relationships that could be construed as a potential conflict of interest.

## Publisher's Note

All claims expressed in this article are solely those of the authors and do not necessarily represent those of their affiliated organizations, or those of the publisher, the editors and the reviewers. Any product that may be evaluated in this article, or claim that may be made by its manufacturer, is not guaranteed or endorsed by the publisher.

## Footnotes

^1^A mediating factor (or mediator) explains the process through which two variables are related and can be a potential mechanism by which the experience of social stress can produce changes in mental health. A moderating factor (or moderator) affects the strength and direction of that relationship.
