# Supplementary material for: Social Inequalities in Young People's Mental Distress During the COVID-19 Pandemic: Do Psychosocial Resource Factors Matter?
Source: Front Public Health. 2022 Mar 14;10:820270. doi: 10.3389/fpubh.2022.820270 (PMC8964111; doi:10.3389/fpubh.2022.820270)
Supplement: Supplementary file 1 [file Table_1.pdf]

Appendix

Table A.1: Summary statistics of the observed exogenous and endogenous variables (N=2,402)

	Mean	Standard deviation	Minimum value	Maximum value
HSCL-5*	0.021	0.735	-1.193	1.691
Future Optimism*	-0.028	0.309	-.609	.484
General Self-efficacy*	0.014	0.470	-1.816	1.099
Social Support	2.485	1.368	0	6
Financial strain	0.444	0.320	0	1
Low parental education (NVQ/ below Level 2/Other)	0.122	0.328	0	1
High parental education (Level 4 or above)	0.456	0.498	0	1
Free School Meal	0.288	0.453	0	1
Employment Status (in education)	0.415	0.493	0	1
Employment Status (NEET)	0.107	0.309	0	1
Living with parents or legal guardian(s)	0.581	0.494	0	1
Male	0.480	0.500	0	1
Age 16-18	0.228	0.420	0	1
Ethnicity (Bame)	0.230	0.421	0	1
Ethnicity (Refusal)	0.028	0.165	0	1
Wave 3 (Jul-21)	0.337	0.473	0	1
Wave 4 (Oct-21)	0.324	0.468	0	1

* predicted latent variable using factor scores.
