# Supplementary material for: Social Inequalities in Young People's Mental Distress During the COVID-19 Pandemic: Do Psychosocial Resource Factors Matter?
Source: Front Public Health. 2022 Mar 14;10:820270. doi: 10.3389/fpubh.2022.820270 (PMC8964111; doi:10.3389/fpubh.2022.820270)
Supplement: Supplementary file 2 [file Table_2.pdf]

Appendix

Table A2: Measurement Models: Correlation between indicators and their prediction (N=2,402)

	Correlation Coefficient	R^2
**General self-efficacy (GSE-6)**		
I find ways to get what I want	0.598	0.357
Stick to aims	0.604	0.365
Resourcefulness	0.693	0.481
Can solve most problems	0.585	0.342
Remain calm	0.695	0.484
Able to handle whatever comes my way	0.721	0.520
**Future optimism**		
Job that pays well	0.675	0.456
Own your own home	0.651	0.424
Job that you enjoy doing	0.659	0.434
Happy family life	0.636	0.404
Good health	0.602	0.362
Respected in your community	0.606	0.367
Good friends	0.529	0.279
Life will turn out better for you than your parents	0.516	0.266
Children will have a better life than you	0.440	0.193
**HSCL-5**		
Feeling fearful	0.797	0.635
Nervousness or shakiness inside	0.793	0.628
Feeling hopeless about the future	0.756	0.571
Feeling blue	0.772	0.595
Worrying too much	0.770	0.593
