# Supplementary material for: Social Inequalities in Young People's Mental Distress During the COVID-19 Pandemic: Do Psychosocial Resource Factors Matter?
Source: Front Public Health. 2022 Mar 14;10:820270. doi: 10.3389/fpubh.2022.820270 (PMC8964111; doi:10.3389/fpubh.2022.820270)
Supplement: Supplementary file 3 [file Table_3.pdf]

Appendix

Table A3: Conditional associations of the control variables with the model endogenous variables (N=2,402)

	Coefficient	Robust std. err.	z	P>|z|
**Financial Strain**				
Employment Status (in education)	0.055	0.025	2.22	0.027
Employment Status (NEET)	0.123	0.023	5.39	0.000
Living with parents or legal guardian(s)	-0.116	0.025	-4.67	0.000
Male	-0.046	0.024	-1.93	0.054
Age 16-18	-0.038	0.024	-1.55	0.121
Ethnicity (Bame)	0.065	0.024	2.74	0.006
Ethnicity (Refusal)	0.038	0.021	1.83	0.067
Wave 3 (Jul-21)	0.007	0.020	0.35	0.723
Wave 4 (Oct-21)	0.011	0.022	0.49	0.621
**Social Support**				
Employment Status (in education)	-0.031	0.026	-1.22	0.223
Employment Status (NEET)	-0.057	0.023	-2.47	0.013
Living with parents or legal guardian(s)	-0.060	0.025	-2.37	0.018
Male	-0.065	0.024	-2.66	0.008
Age 16-18	0.014	0.026	0.56	0.576
Ethnicity (Bame)	0.001	0.023	0.06	0.953
Ethnicity (Refusal)	-0.009	0.024	-0.37	0.713
Wave 3 (Jul-21)	0.005	0.021	0.22	0.826
Wave 4 (Oct-21)	0.044	0.021	2.06	0.040
**General Self-Efficacy**				
Employment Status (in education)	-0.031	0.029	-1.05	0.294
Employment Status (NEET)	-0.087	0.030	-2.87	0.004
Living with parents or legal guardian(s)	-0.062	0.030	-2.09	0.036
Male	0.125	0.028	4.39	0.000
Age 16-18	0.028	0.029	0.98	0.326
Ethnicity (Bame)	0.007	0.029	0.23	0.817
Ethnicity (Refusal)	-0.034	0.025	-1.33	0.184
Wave 3 (Jul-21)	0.006	0.019	0.33	0.740
Wave 4 (Oct-21)	0.005	0.021	0.26	0.798

**Future optimism**				
Employment Status (in education)	-0.068	0.028	-2.44	0.014
Employment Status (NEET)	-0.080	0.024	-3.30	0.001
Living with parents or legal guardian(s)	-0.059	0.027	-2.19	0.029
Male	0.023	0.026	0.88	0.377
Age 16-18	0.085	0.027	3.11	0.002
Ethnicity (Bame)	0.036	0.028	1.29	0.198
Ethnicity (Refusal)	-0.018	0.024	-0.73	0.466
Wave 3 (Jul-21)	-0.007	0.022	-0.31	0.758
Wave 4 (Oct-21)	0.014	0.023	0.61	0.545
**HSCL5**				
Employment Status (in education)	-0.021	0.024	-0.85	0.395
Employment Status (NEET)	-0.034	0.025	-1.38	0.168
Living with parents or legal guardian(s)	-0.095	0.026	-3.66	0.000
Male	-0.183	0.024	-7.55	0.000
Age 16-18	0.086	0.027	3.21	0.001
Ethnicity (Bame)	-0.053	0.023	-2.32	0.020
Ethnicity (Refusal)	0.004	0.018	0.22	0.825
Wave 3 (Jul-21)	0.005	0.019	0.25	0.801
Wave 4 (Oct-21)	-0.011	0.020	-0.57	0.571
